# Attachment, emotion regulation and quality of life in women with a diagnosis of endometriosis: a cross-sectional study

**DOI:** 10.1007/s00404-026-08512-1

**Published:** 2026-07-09

**Authors:** Cristina Sechi, Monica Ahmad, Stefano Guerriero, Silvia Ajossa, Laura Vismara

**Affiliations:** 1 Department of Pedagogy, Psychology, Philosophy, Faculty of Human Studies, University of Cagliari, Cagliari, Italy; 2 Department of Surgical Sciences, Medical School, University of Cagliari, Cagliari, Italy; 3 Department of Surgical Sciences, Azienda Ospedaliera Universitaria Cagliari, Cagliari, Italy

**Keywords:** Endometriosis, Attachment styles, Emotion regulation, Quality of life

## Abstract

**Purpose:**

Endometriosis, a prevalent gynecological condition, significantly impacts physical, psychological, and social well-being. This study explores the relationship between attachment styles, emotion regulation difficulties, and quality of life in women with endometriosis. Drawing on attachment theory, the study hypothesized that secure attachment would correlate with fewer emotion regulation difficulties and higher quality of life, while insecure attachment styles would correlate with greater difficulties and lower quality of life.

**Methods:**

The study included 354 Italian women diagnosed with endometriosis. Participants completed the Relationship Questionnaire to assess attachment styles, the Difficulties in Emotion Regulation Scale—Short Form to evaluate emotion regulation, and the WHOQOL–BREF to measure quality of life. Data analysis employed structural equation modeling to examine the direct and indirect effects of attachment styles on quality of life, mediated by emotion regulation difficulties.

**Results:**

Results confirmed the hypotheses: secure attachment was associated with better emotion regulation and higher quality of life, whereas preoccupied and fearful attachment styles were linked to greater emotion regulation difficulties and lower quality of life. The dismissing attachment style did not show significant associations.

**Conclusions:**

The findings underscore the importance of addressing emotion regulation in therapeutic interventions for women with endometriosis.

## What does this study add to the clinical work

**Table:** 

This study provides evidence that attachment styles significantly influence the quality of life of women with endometriosis, with difficulties in emotion regulation serving as a central mediating mechanism between insecure attachment and poorer psychosocial outcomes. These findings underscore the clinical relevance of incorporating the assessment of attachment patterns and targeted interventions aimed at improving emotion regulation within endometriosis care, in order to enhance patients’ psychological well-being and overall quality of life.	

## Introduction

Endometriosis is the second most common gynecological condition, frequently leading to severe pain, functional impairment, and infertility. Alongside physical symptoms, individuals with endometriosis often face depressive moods and increased anxiety, as well as higher levels of perceived stress, and various types of pain [1–3], all of which affect their social interactions [1]. It also presents considerable psychological burdens, significantly reducing the quality of life for individuals affected [1–3]. Concerns about infertility or subfertility can also contribute to feelings of worry, depression, and inadequacy [4]. Specifically, research has shown that people with endometriosis are at a higher risk for depression, generalized anxiety disorder, and post-traumatic stress disorder [5–7]. Some reviews have demonstrated that endometriosis negatively impacts psychosocial well-being and overall quality of life (QoL) for patients [8, 9]. However, the exact causes of the psychological symptoms associated with endometriosis remain poorly understood. Notoriously, strong social support and the capacity to establish healthy, stable relationships can significantly enhance quality of life, as well as psychological and physical health and well-being [4, 6, 10–12]. This capability can be interpreted through the lens of attachment theory.

Attachment theory posits that newborns are inherently equipped to form attachment relationships with primary caregivers [13, 14]. When caregivers consistently offer sensitive support in response to a child's distress, the child is likely to develop a secure attachment [13]. These interactions are internalized as what Bowlby [13] termed internal working models (IWMs), which are dynamic cognitive frameworks reflecting past experiences and shaping expectations of future support from significant others [15]. Conversely, if caregivers are inconsistent or consistently unavailable, children may develop insecure attachment relationships and insecure IWMs regarding their caregivers' reliability.

Various classifications of attachment have been developed by researchers. A prominent classification in the literature is the model by Bartholomew and Horowitz [16], which divides attachment styles into secure, dismissing, preoccupied, and fearful. 

Secure attachment is characterized by comfort with interdependence and intimacy, and individuals with this attachment style have a balanced view of themselves and others [17]. They also have appropriate expectations of others' responsiveness to their needs [18, 19]. Preoccupied individuals tend to have a negative self-concept and rely on others' approval and affection for self-acceptance [20]. This attachment style is also associated with a fear of rejection or abandonment, leading to increased efforts to seek closeness with others [16].

Dismissive attachment is characterized by an overly positive self-view as independent and self-sufficient, coupled with a negative view of others. This results in an avoidance of intimacy and a downplaying of the importance of relationships [16].

The fearful attachment style is considered a breakdown in attachment strategy, marked by ongoing states of fear and disorganization [14, 15, 20, 21]. Individuals with this attachment style hold a highly negative view of both self and others, desiring closeness yet fearing it intensely, resulting in chaotic and unresolved patterns of intimacy [16, 22].

Literature has shown that individuals' internal working models of relationships influence the capacity for emotion regulation throughout life. Emotion regulation primarily entails assessing stressful events to determine whether they are perceived as threatening and influence the types of emotions individuals experience, as well as when and how these emotions are expressed [17, 23]. Emotion regulation and attachment styles are recognized as crucial for individuals' well-being, influencing their subjective quality of life [18, 24]. Secure attachment and emotional regulation are positively linked with higher levels of well-being, whereas insecure attachment styles and emotional dysregulation are associated with lower levels of quality of life [19, 20, 25, 26].

These variables seem crucial in understanding the psychological context of endometriosis: reduced ability in emotion regulation may heighten the occurrence of negative emotions, and uncontrolled negative emotions can exacerbate persistent pain, creating a mutually reinforcing cycle, and decreasing these women’s quality of life [21–23, 27–29].

Indeed, several studies on chronic illness have shown an association between attachment and chronic illness [30–33], highlighting poorer parental care and more insecure attachment in these populations compared to non-clinical samples. However, significant differences between patients with different chronic medical conditions should be considered [25, 34].

Given the diffusion of endometriosis and the aforementioned impact on their quality of life, it is crucial to have a clear understanding of the typical characteristics of most patients with such diagnosis.

For this reason, the current study aimed to enhance our understanding of attachment styles and their connection with emotion regulation difficulties and quality of life in individuals diagnosed with endometriosis. Specifically, our hypotheses were:

- a significant negative relationship between secure attachment style and emotion regulation difficulties, as well as a positive relationship with high quality of life;
- a significant relationship between insecure attachment styles, emotion regulation difficulties, and diminished quality of life.

## Materials and methods

### Participants

The study included 354 women with a physician-diagnosed endometriosis. Psychiatric diagnoses were assessed using self-report measures and referred to both current and lifetime conditions. Participants' ages ranged from 18 to 64 years, with a mean age of 37.30 years (SD = 7.47). The average time since diagnosis was 5.14 years (SD = 4.64), with a range of one to 15 years. Most participants were married (61%), while 31.4% were single, 7.6% were separated/divorced. Regarding education, 41% held a university degree, 36.4% had a high school diploma or equivalent, 12.1% had a postgraduate degree, and 10.5% had a middle school diploma. Geographically, 36.7% of participants were from Southern Italy and the Italian Islands, 17.2% from Central Italy, and 46% from Northern Italy. In terms of employment, the majority (88.4%) were actively working, while 41 participants (11.6%) were students or trainees.

No incentives were offered for participation. The sampling method was non-probabilistic, involving women who voluntarily participated in the research.

### Measures

Adult attachment styles: We utilized the Relationship Questionnaire (RQ; [10, 16] to evaluate adult attachment styles. This instrument consists of four paragraphs that describe secure, dismissing, preoccupied, and fearful attachment styles. Participants rated themselves on each attachment style using a 7-point scale ranging from 1 (strongly disagree) to 7 (strongly agree).

The RQ is widely employed as a measure of adult attachment and has demonstrated satisfactory validity through concurrent assessments of interpersonal functioning based on self-reports and reports from friends [160]. In addition, longitudinal studies using observer-based assessments have further supported its validity in understanding personality and behavioral characteristics over time [24, 35]. The RQ is commonly utilized within the general adult population experiencing chronic pain [26, 33, 36].

Emotion regulation difficulties: We employed the Difficulties in Emotion Regulation Scale—Short Form (DERS-SF) [25, 26, 37, 38] to assess emotion regulation difficulties. This 18-item shortened version is adapted from the comprehensive DERS [27, 28, 39, 40]. The DERS-SF evaluates six domains of emotion regulation difficulties: (A) lack of emotional awareness (AW), (B) lack of emotional clarity (CLA), (C) challenges in goal-directed behavior (GO), (D) difficulties with impulse control (IMP), (E) non-acceptance of emotional responses (NAP), and (F) strategies for regulating emotions (STR). Responses are recorded on a 5-point Likert scale, ranging from 1 ("almost never") to 5 ("almost always"), with higher scores indicating more pronounced difficulties in each domain. As with the original DERS-SF, an overall or total score is computed to provide a comprehensive assessment. The DERS is commonly utilized within the general adult population experiencing chronic pain [41, 42].

Quality of life: We employed the World Health Organization Quality of Life Questionnaire [29, 30, 43, 44] to measure participants’ quality of life. This scale comprises 26 items rated on a 5-point Likert scale (ranging from 1, "not at all," to 5, "completely"). The WHOQOL–BREF assesses quality of life across four domains: physical health (PHY, 7 items), psychological health (PSY, 6 items), social relationships (SR, 3 items), and environment (EN, 8 items). In addition, two items provide an overall assessment of QoL/health.

The WHOQOL–BREF has been widely used to evaluate quality of life in various populations, including individuals with conditions like endometriosis [31, 45, 46]. Higher scores indicate higher perceived quality of life. Numerous studies across different countries have demonstrated good internal consistency, reliability and construct validity for the international version of the WHOQOL–BREF. The WHOQOL–BREF is commonly utilized within the general adult population experiencing chronic pain disease [26, 33, 47–49].

Socio-demographic and clinical data related to endometriosis were investigated through questions placed in the final section of the battery of tools. The age of participants, their geographical origin, employment status, and marital status were reported. Regarding the clinical characteristics of our sample, participants were asked how many years they had been experiencing endometriosis symptoms and how many years it had been since the official diagnosis was made. This way, the diagnostic delay was also determined.

### Recruitment and procedure

Participants in this study were women diagnosed with endometriosis, irrespective of whether they were undergoing surgical treatment or managing their condition with medication. Inclusion criteria specified participants had to be at least 18 years and have a certified diagnosis of endometriosis for at least 1 year. Exclusion criteria included the presence of a psychiatric diagnosis. Recruitment involved collaboration with the "Integrated Center for Assisted Reproduction and Obstetric-Gynecological Diagnosis" of the University Hospital of Cagliari; we also contacted women through three associations: the “Endometriosis Project Association” (APE), “One Voice is Everyone’s Voice odv,” and “A.l.i.c.e. odv.”(Italian Association for Endometriosis Awareness and Struggle), nonprofit organizations, that agreed to disseminate the questionnaire among the women affiliated with them.

All participants received a web-based questionnaire detailing the study’s objectives, emphasizing voluntary participation, and including an informed consent form. The research received approval from the Ethics Committee of the University of Cagliari, Prot. n. 0230694 10/10/2023. Participants received the questionnaires via email and were contacted personally by the study authors or through collaborating associations. The email specified the research objective and included the contact information of the researchers responsible for the study. It also contained a clickable link that opened a web-based questionnaire, which could be filled out online.

### Data analyses

We conducted statistical analyses using SPSS and AMOS 21. Initially, the data underwent rigorous screening for errors and outliers. For handling missing data, we employed multiple imputation (MI) [33, 50]. Before applying MI, we assessed the missing data to ensure they met the missing at random (MAR) assumption and verified that less than 10% of data were missing across scale scores. Subsequently, we generated 25 multiply imputed datasets.

We explored the distribution of all study variables and performed descriptive and correlation analyses. The level of significance (alpha) was set at a 0.05.

After establishing correlations among the variables, we conducted mediation tests using a structural equation model (SEM) to examine whether emotion regulation difficulties mediate the relationship between attachment styles and quality of life (QoL). The model included four observed independent variables—attachment styles measured by the Relationship Questionnaire (RQ) and operationalized as four independent continuous ratings on a 7-point Likert scale—a latent mediating factor (DERS), and a latent dependent factor representing quality of life (QoL). The DERS latent factor was assessed using the six subscales of the DERS-SF (AW, CLA, GO, IMP, NAP, STR), while the QoL latent factor was measured using the four subscales of the WHOQOL–BREF (PHY, PSY, SR, EN). To evaluate the hypothesized model, we considered several fit indices [34, 51]. Given the sensitivity of the *χ*^2^ statistic to sample size, we also examined additional fit indices: the comparative fit index (CFI), the Tucker Lewis Index (TLI), the standardized root-mean-square residual (SRMR), and the root-mean-square error of approximation (RMSEA), including its 90% confidence interval (CI). In general, CFI and TLI values above 0.95 and SRMR and RMSEA values below 0.08 are indicative of good model fit [25, 52]. We employed a bootstrapping procedure with 1500 subsamples to assess the statistical significance of each path coefficient.

## Results

### Preliminary analysis

No statistically significant associations were observed between years since diagnosis and the study variables. Table 1 displays descriptive statistics, including means and standard deviations, as well as correlations among all variables in the model. At the bivariate level, confirming our hypothesis (hypothesis 1), secure attachment style showed a negative correlation with emotion regulation difficulties and positive correlations with physical, psychological, social, and environmental health.

**Table 1:** Descriptive statistics and inter-scale correlations

	M	SD	1	2	3	4	5	6	7	8	9	10	11	12	13	14
1.SA	2.49	1.87														
2.DA	3.24	2.00	– 0.07													
3.PA	2.85	1.77	– 0.13*	0.01												
4.FA	3.65	2.00	– 0.19***	0.02	0.44***											
5. AW	8.61	3.43	– 0.27***	0.00	0.14**	0.10										
6.CLA	7.65	2.97	– 0.27***	– 0.09	0.17**	0.18**	0.49***									
7.GO	8.47	3.55	– 0.36***	0.01	0.27***	0.28***	0.50***	0.53***								
8.IMP	9.78	3.40	– 0.31***	– 0.06	0.22***	0.22***	0.53***	0.40***	0.51***							
9.NAP	6.82	3.45	– 0.26***	0.07	0.19***	0.15**	0.50***	0.44***	0.58***	0.54***						
10.STR	8.75	3.37	– 0.36***	– 0.08	0.31***	0.28***	0.44***	0.53***	0.61***	0.68***	0.62***					
11.PHY	2.74	0.75	0.28***	0.10	– 0.22***	– 0.25***	– 0.34***	– 0.38***	– 0.39***	– 0.50***	– 0.24***	– 0.41***				
12.PSY	2.77	0.69	0.50***	0.09	– 0.26***	– 0.21***	– 0.37***	– 0.42***	– 0.50***	– 0.50***	– 0.35***	– 0.51***	0.64***			
13.SR	2.77	0.64	0.26***	0.02	– 0.12*	– 0.17**	– 0.32***	– 0.32***	– 0.35***	– 0.33***	– 0.17**	– 0.28***	0.63***	0.66***		
14.EN	2.78	0.80	0.42***	0.03	– 0.27***	– 0.23***	– 0.20***	– 0.31***	– 0.28***	– 0.34***	– 0.21***	– 0.37***	0.44***	0.58***	0.38***	

Adult attachment styles (*SA* = Secure Attachment Style; *DA* = Dismissing Attachment Style; *PA* = Preoccupied Attachment Style; *FA* = Fearful Attachment Styles); Emotion regulation difficulties (*AW* = lack of emotional awareness; *CLA* = lack of emotional clarity; *GO* = challenges in goal-directed behavior; IMP = difficulties with impulse control; *NAP* = non-acceptance of emotional responses; *STR* = strategies for regulating emotions); Quality of life (*PHY* = Physical Health; *PSY* = Psychological Health; *SR* = Social Relationships; *EN* = Environment)

***p < 0.001; ** *p* < 0.01; *p <0 .05

In contrast, preoccupied and fearful attachment styles were linked to elevated levels of emotion regulation difficulties and poorer physical, psychological, social, and environmental health (hypothesis 2). Dismissing attachment styles did not exhibit significant associations with emotion regulation difficulties or quality of life, contrary to our hypothesis. Furthermore, emotion regulation difficulties showed negative associations with physical, psychological, social, and environmental health.

### Tests of mediation

Preliminary bivariate correlation analyses (see Table 1) indicated no statistically significant associations between the dismissing attachment style and the outcome variables; therefore, this attachment dimension was excluded from subsequent multivariate models. Similarly, years since diagnosis was not included as a covariate in the SEM analyses, as it showed no statistically significant associations with the primary study variables. These exclusions were implemented to ensure model parsimony, limit unnecessary model complexity, and avoid the inclusion of predictors that did not meaningfully contribute to the explanatory power of the model. Specifically, the model assessed the direct impact of each attachment style (secure, preoccupied, and fearful) on emotion regulation difficulties, as well as the direct and indirect effects of each attachment style (secure, preoccupied, and fearful) on QoL. In addition, they examined the direct effect of emotion regulation difficulties on QoL.

**Fig. 1:**
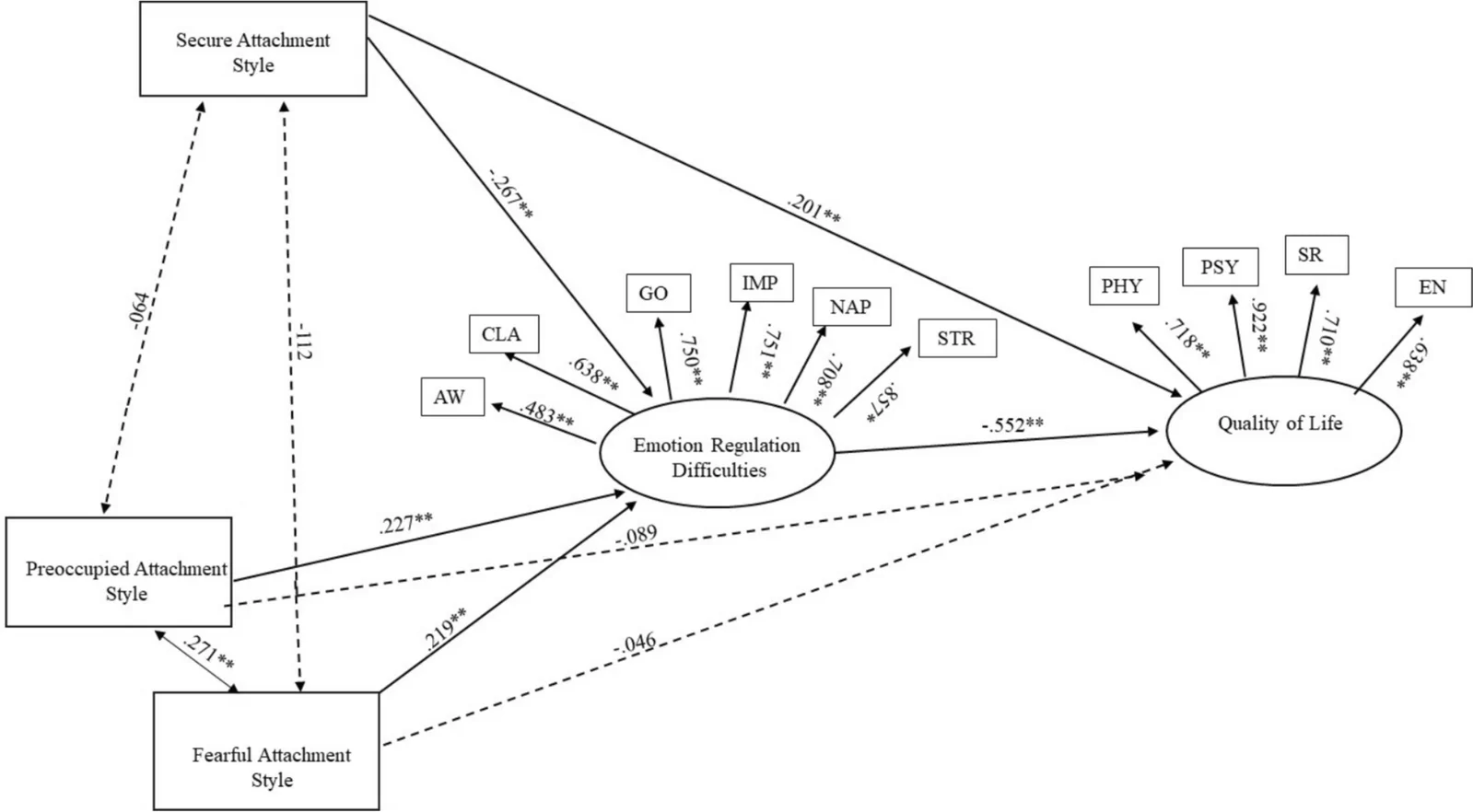
Structural model with standardized path coefficients. The dashed line indicates non-significant path. *AW* lack of emotional awareness; *CLA* lack of emotional clarity; *GO* challenges in goal-directed behavior; *IMP* difficulties with impulse control; *NAP* non-acceptance of emotional responses; *STR* strategies for regulating emotions; *PHY* physical health; *PSY* psychological health; *SR* social relationships; *EN* environment. **p < 0.001

Table 2 reports the standardized direct and indirect effects derived from the mediation model. Figure 1 visually depicts these relationships for each attachment style, emotion regulation difficulties, and QoL.

**Table 2:** Standardized direct and indirect effects

Attachment styles	Emotion regulation difficulties	QoL	
	Direct	Direct	Indirect
Secure attachment style	– 0.267* CI = – 0.347 to – 0.180	0.201* CI = 0.113 to 0.283	0.147* CI = 0.097 to 0.197
Preoccupied attachment style	0.0.227* CI = 0.140 to 0.311	– 0.089 CI = – 0.171 to – 0.010	– 0.125* CI = – 0.181 to – 0.078
Fearful attachment style	0.219** CI = 0.131 to 0.300	– 0.046 CI = – 0.120 to 0.040	– 0.121* CI = – 0.172 to – 0.075

The confidence intervals (CI) are based on the findings from bootstrapping analysis (1500 samples)

*p < 0.01

### Structural model

The structural equation model (SEM) was estimated using robust maximum likelihood estimation (MLR) to account for potential departures from multivariate normality. Overall model fit was evaluated using multiple fit indices, which indicated an acceptable but suboptimal fit to the data (*χ*^2^ = 251.84, df = 58, *p* < 0.001; CFI = 0.90; IFI = 0.91; RMSEA = 0.08, 90% CI [0.05, 0.11]; SRMR = 0.05). Although the model demonstrated overall adequacy, several fit indices fell within the borderline range. Specifically, the CFI and IFI values approached but did not reach the more conservative recommended threshold of 0.95, and the RMSEA value was at the upper limit of acceptability. These results suggest that while the model provides a reasonable approximation of the observed data, it may not fully capture the complexity of the relationships among the study variables.

As shown in Fig. 1, all standardized factor loadings were statistically significant, providing evidence for the adequacy of the measurement model.

The explained variance of the endogenous latent variables was assessed using squared multiple correlations (SMC) as provided by AMOS 21. The model accounted for 18% of the variance in emotion regulation difficulties (*R*^2^ = 0.18) and 48% of the variance in quality of life (*R*^2^ = 0.48).

At the structural level, secure attachment style exhibited a significant negative direct effect on emotion regulation difficulties and significant direct and indirect effects on quality of life (QoL). Emotion regulation difficulties, in turn, showed a significant direct effect on QoL. The model explained a substantial proportion of variance in the latent mediator (emotion regulation difficulties) and in the latent outcome (QoL), as indicated by the respective *R*^2^ values. Collectively, these results support a partial mediation model in which secure attachment influences QoL both directly and indirectly through its effect on emotion regulation processes. There was no significant direct effect of Preoccupied Attachment Style on QoL. However, preoccupied attachment style significantly affected emotion regulation difficulties, which in turn had a significant direct effect on QoL. These results suggest that preoccupied attachment style indirectly affects QoL through emotion regulation difficulties. Women with endometriosis who exhibit higher levels of preoccupied attachment are more likely to face greater emotion regulation difficulties, thereby lowering their QoL.

Similarly, Fearful Attachment Style had no significant direct effect on QoL but did have a significant indirect effect through emotion regulation difficulties. Fearful Attachment Style significantly impacted emotion regulation difficulties, which then significantly affected QoL. These findings show that fearful attachment style indirectly influences QoL through emotion regulation difficulties. Women with endometriosis who display higher levels of fearful attachment are more prone to experiencing greater difficulties in emotion regulation, which may subsequently reduce their QoL.

## Conclusions and discussion

The present study aimed to examine the associations among attachment styles, emotion regulation difficulties, and quality of life in women living with endometriosis. Overall, the findings suggest that insecure attachment styles—particularly preoccupied and fearful attachment—are associated with greater difficulties in emotion regulation and lower levels of perceived quality of life. Consistent with the hypothesized model, emotion regulation difficulties statistically mediated the relationship between attachment orientations and quality of life. These results align with attachment theory, which proposes that internal working models of relationships shape individuals’ ability to regulate emotional experiences and cope with stressful life circumstances, including chronic illness.

Specifically, there is substantial evidence indicating that preoccupied attachment influences health and symptom experiences, such as stroke, heart attack, high blood pressure, ulcers, arthritis, psoriasis, migraine, fibromyalgia, and cancer survivors. Actually, preoccupied women tend to perceive stressful events as more threatening, possess a lower resilience to stress, and employ coping strategies that focus on their own emotional distress, thereby exacerbating their reduced quality of life [30, 33, 53–55]. On the other hand, individuals with fearful attachment styles often seek assistance only when experiencing extreme distress. They may exhibit a sense of hopelessness and a reluctance to let go of their pain and distress [35, 56].

Unexpectedly, no significant association was observed for dismissing attachment. Although dismissive attachment has been consistently linked to maladaptive emotion regulation, lower well-being, and increased anxiety and depression [17, 57, 58], this pattern did not emerge in the present study. One tentative explanation is that the positive self-image characteristic of dismissing individuals may act as a short-term resilience factor, buffering perceived quality of life despite chronic illness. In addition, a negative view of others may reduce the subjective salience of interpersonal difficulties related to endometriosis, thereby attenuating their impact on reported quality of life. Avoidant and deactivating strategies may also provide temporary psychological distance, allowing time for adjustment and processing, with potential short-term benefits [36, 59]. However, alternative explanations related to measurement issues must be considered. Individuals with dismissing attachment tend to minimize emotional distress and may under-report difficulties on self-report measures, potentially obscuring associations with quality of life. Moreover, the relationship questionnaire may be limited in its ability to capture defensive processes and implicit distress characteristic of dismissing attachment. Therefore, the absence of significant findings should be interpreted with caution and does not necessarily indicate the absence of psychological impact. Future research employing longitudinal designs, multi-method assessments, and measures sensitive to deactivating strategies is needed to clarify the role of dismissing attachment in adjustment to endometriosis.

Conversely, secure attachment forms a strong foundation for enhancing quality of life. As has been highlighted by previous studies, a secure attachment style is notably associated with high psychological functioning, effective coping strategies, and resilience [37–39, 60–62]; in fact, people with a secure attachment style use emotional regulation strategies that minimize stress and activate positive emotions [63, 64],

Moreover, in line, our study’s tested models suggest that emotion regulation mediates the relationship between attachment and quality of life. This complex and multifaceted process involves understanding emotions, managing them effectively, and developing self-awareness. As previous studies have pointed out, improved emotion regulation is associated with higher life satisfaction, protection against psychopathology, increased workplace effectiveness, and the ability to form lasting adult relationships [40, 41, 65, 66]. Emotion regulation, which involves experiencing, processing, and adjusting emotional responses, is essential for handling the emotional challenges often faced by patients with chronic illnesses. Strengthening emotion regulation skills helps individuals adapt to stressful situations, while poor management of emotions triggered by a health condition can undermine self-care efforts and negatively affect both mental and physical health [67]. It is plausible that women with poorer emotion regulation may feel overwhelmed by life problems, perceiving them as unmanageable and beyond their control.

Although the tested mediation model was theoretically grounded in attachment theory and supported by prior empirical evidence, it is important to acknowledge that alternative conceptual models are also plausible. Given the cross-sectional nature of the data, the directionality of the observed associations cannot be definitively established. For instance, reduced quality of life associated with chronic pain and functional impairment in endometriosis may itself undermine emotion regulation capacities over time, rather than emotion regulation solely predicting quality of life. Similarly, persistent difficulties in regulating emotions may influence how individuals perceive and report attachment-related patterns in close relationships, particularly under conditions of chronic stress and illness. These reciprocal or bidirectional processes are consistent with transactional models of psychological adjustment in chronic disease and should be examined in future longitudinal research. Such designs would allow for a more nuanced understanding of the dynamic interplay among attachment, emotion regulation, and quality of life.

### Limitations and future studies

Although this study revealed valuable insights into the predictive validity of adult attachment styles in relation to emotion regulation difficulties and quality of life (QoL) among women with endometriosis, these findings should be interpreted with caution due to several limitations.

Although the structural equation model demonstrated an acceptable fit to the data, several fit indices fell within borderline ranges, suggesting that the model may not fully capture the complexity of the relationships among attachment, emotion regulation, and quality of life. Furthermore, the model explained a moderate proportion of variance in quality of life but a smaller proportion of variance in emotion regulation difficulties. This pattern indicates that additional psychological, interpersonal, and clinical variables may also contribute to these outcomes and should be considered in future research.

Importantly, the cross-sectional design of this study precludes establishing causality or the directionality of relationships. Undeniably, further research is essential to deepen our understanding of the connections between attachment, emotion regulation and health outcomes. Although the proposed model assumes that attachment influences emotion regulation processes, which in turn relate to quality of life, alternative explanations are also plausible. For instance, the chronic pain and functional impairment associated with endometriosis may themselves undermine emotion regulation capacities over time. Similarly, reduced quality of life may influence individuals’ perceptions of interpersonal relationships and attachment-related experiences. These possibilities are consistent with transactional models of psychological adjustment in chronic illness and highlight the need for longitudinal studies capable of examining reciprocal and dynamic relationships among these variables.

Despite these limitations, the study contributes to the growing literature on the psychological dimensions of endometriosis by integrating attachment theory with emotion regulation frameworks. While previous research has documented elevated levels of psychological distress and reduced quality of life among women with endometriosis, relatively few studies have explored relational and developmental factors that may shape emotional adjustment in this population. By examining attachment orientations alongside emotion regulation difficulties, the present research offers a more comprehensive perspective on psychological vulnerability and resilience in women coping with this chronic condition.

From a clinical perspective, the findings underscore the potential relevance of psychosocial processes in the management of endometriosis. Understanding how attachment-related patterns influence emotional responses to illness may help healthcare professionals identify patients who are particularly vulnerable to psychological distress. Interventions aimed at strengthening emotion regulation skills and promoting adaptive coping strategies may, therefore, represent promising avenues for psychosocial support. However, further longitudinal and intervention-based research is needed to clarify whether targeting these processes can effectively improve psychological well-being and quality of life among women with endometriosis.

An additional limitation concerns the clinical heterogeneity of endometriosis. The present study did not include information on disease stage, pain severity, duration of symptoms, or ongoing medical or surgical treatments. These factors are known to vary substantially among women with endometriosis and may independently or interactively influence emotion regulation capacities and perceived quality of life. For example, higher pain severity or advanced disease stages may exacerbate emotional distress and impair adaptive coping, potentially confounding the observed associations. Likewise, different treatment regimens (e.g., hormonal therapy, surgery, or pain management strategies) may affect both psychological functioning and quality of life. The absence of these clinical variables limits the ability to disentangle psychological from disease-related contributors to quality of life and should be addressed in future studies through more comprehensive clinical assessments.

Moreover, adverse childhood experiences, such as neglect, abuse, or inconsistent caregiving behaviors, can impact attachment development and heighten the likelihood of developing both insecure attachment styles and trauma-related symptoms. Therefore, it is considered crucial to assess early experiences in research to identify individuals who are particularly at risk of struggling to adapt to the various challenges associated with chronic diseases, such as endometriosis. Given the high prevalence of psychiatric comorbidity among women with endometriosis, the exclusion of participants on the basis of psychiatric criteria in the present study represents a significant source of selection bias. As a result, the study sample may not be fully representative of the broader population of women with endometriosis, thereby potentially limiting the external validity and generalizability of the findings to real-world clinical settings in which psychiatric comorbidities are common.

In addition, all measures relied on self-reported data, potentially introducing social desirability bias.

In particular, the use of the Relationship Questionnaire (RQ) to assess adult attachment represents an additional methodological consideration. While the RQ is a widely used and efficient instrument, it provides a relatively coarse assessment of attachment patterns compared with more fully dimensional measures of adult attachment (e.g., instruments assessing anxiety and avoidance on continuous scales). Consequently, the use of the RQ may limit the granularity with which individual differences in attachment are captured and could have influenced the observed associations.

Utilizing alternative measures of attachment, such as the Adult Attachment Interview [42, 68] could enhance the accuracy and reliability of attachment style assessments.

In conclusion, the present study highlights the importance of considering relational and emotional processes when examining psychological adjustment in women living with endometriosis. Attachment orientations appear to be associated with differences in emotion regulation capacities, which in turn are linked to perceived quality of life. Recognizing these psychosocial dimensions may encourage a more integrative approach to endometriosis care, moving beyond a purely biomedical perspective toward a more comprehensive biopsychosocial understanding of the condition.

## Data Availability

The data presented in this study are available on request from the corresponding author due to privacy.
